# SEOpinion: Summarization and Exploration of Opinion from E-Commerce Websites

**DOI:** 10.3390/s21020636

**Published:** 2021-01-18

**Authors:** Alhassan Mabrouk, Rebeca P. Díaz Redondo, Mohammed Kayed

**Affiliations:** 1 Mathematics and Computer Science Department, Faculty of Science, Beni-Suef University, Beni Suef 62511, Egypt; alhassanmohamed@science.bsu.edu.eg; 2 Information & Computing Lab, AtlanTTIC Research Center, Telecommunication Engineering School, Universidade de Vigo, 36310 Vigo, Spain; rebeca@det.uvigo.es; 3 Computer Science Department, Faculty of Computers and Artificial Intelligence, Beni-Suef University, Beni Suef 62511, Egypt

**Keywords:** sentiment analysis, hierarchical aspect-based opinion summarization, web scraping, BERT, deep learning techniques

## Abstract

Recently, it has been found that e-commerce (EC) websites provide a large amount of useful information that exceed the human cognitive processing capacity. In order to help customers in comparing alternatives when buying a product, previous research authors have designed opinion summarization systems based on customer reviews. They ignored the template information provided by manufacturers, although its descriptive information has the most useful product characteristics and texts are linguistically correct, unlike reviews. Therefore, this paper proposes a methodology coined as SEOpinion (summarization and exploration of opinions) to summarize aspects and spot opinion(s) regarding them using a combination of template information with customer reviews in two main phases. First, the hierarchical aspect extraction (HAE) phase creates a hierarchy of aspects from the template. Subsequently, the hierarchical aspect-based opinion summarization (HAOS) phase enriches this hierarchy with customers’ opinions to be shown to other potential buyers. To test the feasibility of using deep learning-based BERT techniques with our approach, we created a corpus by gathering information from the top five EC websites for laptops. The experimental results showed that recurrent neural network (RNN) achieved better results (77.4% and 82.6% in terms of F1-measure for the first and second phases, respectively) than the convolutional neural network (CNN) and the support vector machine (SVM) technique.

## 1. Introduction

E-commerce (EC) (e.g., Amazon, eBay, Flipkart, and Snapdeal) websites support the quick expansion of consumer reviews and feedback [1,2]. These reviews are valuable to both product developers (marketers) and consumers. Product developers are interested in identifying those aspects (attributes) that are important for consumers. Additionally, consumers arguably make purchase decisions based on previous evaluations from other customers. With the development of EC websites, popular products (e.g., iPhone and Samsung) contain large amounts of text reviews. Thus, summarizing the aspects of these products is a complex and time-consuming process [3]. Hence, it is essential to provide an aspect-based opinion summarization (AOS) [4]. This paper improved AOS using deep learning (DL)-based BERT [5] embedding. Recently, DL technologies have been used with big data analytics. Everywhere, big data are part of many information processing systems like science, government, health care [6], security/privacy, finance [7], and social media [8].

AOS is a sentiment analysis task that can summarize opinions on aspects given a set of reviews. This task always involves three phases [9]: (i) aspect extraction, (ii) aspect-level polarity detection, and (iii) summary presentation. First, the aspect extraction phase fetches the topics from the review text [10]. For example, a sentence such as “The screen of my laptop is nice and its resolution is good” has two aspects, namely the “screen” and the “resolution.” Second, the aspect-level polarity detection phase determines the sentiment orientation (positive or negative) on the extracted aspects. In the above example, the sentence has two positive aspects: “screen” and “resolution.” The two previous phases of automatic aspect extraction and polarity/strength prediction are jointly called aspect-based sentiment analysis in which more information is provided [11]. Finally, in the summary presentation phase, the processed results are presented by aggregating polarity ratings for all aspects and summarizing opinions around them.

The extracted aspects in AOS systems are represented using two different structures: a flat structure and a hierarchical structure. Using the flat structure means the aspects of a specific domain are represented as a list [12]. For example, a laptop is represented as a list of the two aspects “screen” and “resolution” in the example above. On the other hand, using the hierarchical structure means the aspects of a specific domain are structured into a multi-granularity of aspects [13]. For example, the hierarchical structure of the same example has two levels in addition to the root (“laptop”), in which the “screen” (level 1) is the aspect of a laptop and the “resolution” (level 2) is the aspect of the “screen.” Most approaches of AOS ignore the hierarchical structure inside the aspects [14,15] However, it is very valuable to buyers and product manufacturers in quickly understanding the accurate aspects of massive amounts of consumer reviews, in addition to the hierarchical nature of aspect terms. Alternatively, few researchers have attempted to summarize the opinions of multi-granular aspects, which seems to be more appropriate than flat aggregation [16].

**Figure 1:**
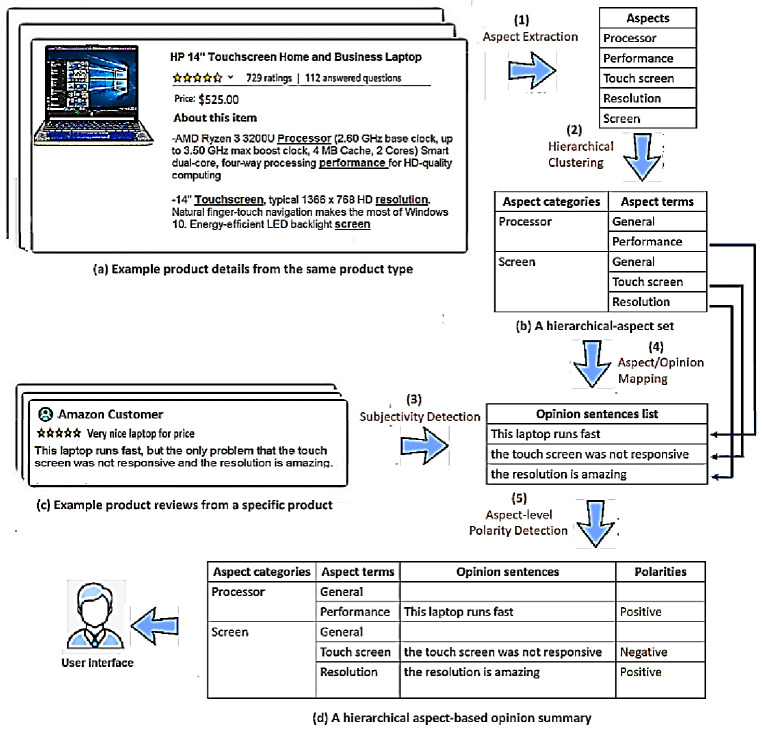
The steps of the proposed approach: SEOpinion.

Hierarchical aspects-based opinion summarization is a challenging problem [16], especially when an aspect hierarchy is provided manually (i.e., a predefined structure) [13] or a large amount of training data are needed for a summary presentation [17]. These problems mean the current hierarchical AOS approaches are not scalable. Thus, this paper proposes an automated approach called SEOpinion that extracts popular aspects from the product details (i.e., the templates of the websites) in a hierarchical structure and classifies the opinionated sentences (from customer reviews) according to their aspects. Our proposed approach includes five main tasks (see Figure 1). In the first task, the aspects are extracted from a set of products (e.g., HP, Dell, and Apple) of the same product type (e.g., laptop), as shown in Figure 1a. The second task constructs an aspect hierarchy using the extracted aspects. The third task extracts opinionated sentences from the product reviews. The fourth task automatically maps the aspects in the hierarchy to extracted opinion sentences. For instance, the opinionated sentence “This laptop is ok for its price” matches the aspect “price.” The fifth task classifies the sentiment polarity (positive or negative) of each opinionated sentence associated with its aspect to be ready for summarization.

The advantages of our SEOpinion system are: (i) it helps users to easily access a sentiment score about any preferred aspect and all sub-aspects thereof; (ii) the extracted aspects do not depend on a specific domain (e.g., Amazon and Flipkart) as long as these aspects are directly extracted from the site templates; (iii) the extracted hierarchy of aspects is constant and does not change with the change of reviews as in other methods [18,19] because they have been obtained from product details, not from the reviews (as shown in step 1 of Figure 1); (iv) some users might prefer to read the actual opinion sentences instead of reading the overall statistics, so these are displayed in a separate panel called the opinion sentence exploration (the details are shown in Section 3.5); (v) it allows users to easily compare people’s opinions on products of the same type (e.g., cameras) because they are all represented by the same aspects (e.g., Zoom, Lens, and Focus); and finally (vi) it helps in the polarity classification processes, which show the polarities of some sentiment words. For example, the sentence “In this laptop, the processor and battery-life are fast” contains a sentiment word “fast” and the two aspects “processor” and “battery-life.” The “fast” is positive under the “processor” aspect node, while it is negative under “battery-life.”

To the best of our knowledge, no prior works have focused on summarizing the opinions of hierarchical aspects extracted from product details. Thus, our main contributions are as follows:

1. Create a web scraper to crawl the product details and reviews from e-commerce websites using XPath (XML path language).
2. Construct a hierarchy of the relevant product aspects that are obtained from the product details and descriptions published in the web pages by the manufacturers.
3. Map each review sentence directly to its corresponding aspect in the hierarchy. Thus, for each product aspect, the sentiment-score and opinionated sentences are shown.
4. Create a corpus, which is obtained from the top five EC (laptops) websites, to validate the proposed approach.
5. Our results showed that the usage of BERT [5] embedding in a recurrent neural network (RNN) model gave better results than convolutional neural network (CNN) and support vector machine (SVM) on our corpus.

The rest of the paper is organized as follows. Section 2 presents related works about aspect-based opinion summarization. The proposed system is discussed in Section 3. Section 4 shows the details of the experiment. The results of our experiment and their analysis are given in Section 5. A discussion of the limitations of our proposed system, as well as future directions, is provided in Section 6. Finally, Section 7 concludes our work.

## 2. Related Works

Most studies have been devoted to performing an opinion summarization task. Wu et al. [4] produced an opinion summarization approach according to emotion modeling. Yang et al. [20] generated text summarization with good grammar using an adversarial network. Yang et al. [21] proposed a novel hierarchical human-like deep neural network for improving the performance of abstractive text summarization. However, these methods ignored the AOS task. In contrast, Bahrainian and Dengel [14] used a hybrid polarity detection method in order to summarize aspects of multiple documents. They used topic detection algorithms to discover different domain-based lexicons. Their algorithm recognized newly added features but was less accurate than manual detection. Zhu et al. [12] suggested a framework for summarizing an opinion based on a sentence. Additionally, they assessed each review’s helpfulness while considering coverage and frequency. Jmal and Faiz [15] introduced the aspect summary approach for an entire product by calculating a score between 0 and 1 for its characteristics based on adverbs, nouns, verbs, and adjectives. However, these mentioned methods extracted their aspects in a flat structure without considering the natural hierarchical structure inside the aspects.

Few methods have addressed the hierarchical structure of the aspects to be extracted. In a sentiment analysis, Kim et al. [22] proposed an unsupervised the hierarchical aspect sentiment model (HASM) to discover a hierarchical structure of aspect summarization from unlabeled online reviews. HASM deals aspect extraction with sentiment modeling. Almars et al. [23] proposed hierarchically modelling users’ interests and sentiments on various topic levels in a tree. However, these models were proposed for aspect identification, and their effectiveness were not investigated for sentiment summarization.

Some researchers have targeted summarizing opinions of multi-granular aspects, which are more appropriate than flat aggregation due to the hierarchical nature of the aspect terms. Pavlopoulos and Androutsopoulos [16] introduced a domain-independent method to group aspects. They also investigated word-vectors based on WordNet to calculate the similarities between words in the hierarchical clustering algorithm. However, some problems, such as manually generated, domain-specific, or pre-defined ontology trees, have been identified in the existing hierarchical aspect-based summarization systems.

Recent research work on hierarchical aspect aggregation [18] proposed an automatic approach to generate an aspect ontology tree using similarity techniques that worked across domains. They considered WordNet in aspect aggregation because word embedding could overcome such limitations encountered in WordNet. In contrast, in [24], the authors built a hierarchy using word embedding to represent each aspect by a vector and then clustering those vectors. However, these mentioned methods are distinct taxonomies that can be generated for two products of the same type. On the other hand, OpinionLink enriches the product aspects, which were designed by human readers, with opinions extracted from user reviews, as proposed in [19]. In contrast, our system automatically discovers product aspects using information from the webpage templates.

In order to enhance the results of the aspect extraction process, some approaches have exploited product reviews in addition to the terms embedded in the web page template of the product. Park et al. [25] used the structure list of the template to extract aspects. However, getting structured product specifications is very expensive. In contrast, in [26], the authors incorporated customer reviews and product descriptions, that are provided by the manufacturers. However, to the best of our knowledge, these methods do not address/show the effect of applying these extracted aspects from page’ templates on the opinion summarization problem. Therefore, this paper addresses the problem of mapping opinionated sentences with the extracted hierarchical-aspects from templates.

To address some of these shortcomings, we introduce a novel approach that produces aspect-based opinion summaries by merging product details with customer reviews. Our system will be presented in details below.

## 3. SEOpinion: Methodology

This section describes the proposed SEOpinion system in detail. The first subsection gives an overview of the system architecture. Afterward, the scraping process, hierarchical aspect extraction (Phase A), and hierarchical aspect-based opinion summarization (Phase B) are addressed. Lastly, the SEOpinion’s interface is discussed.

### 3.1. Overview

**Figure 2:**
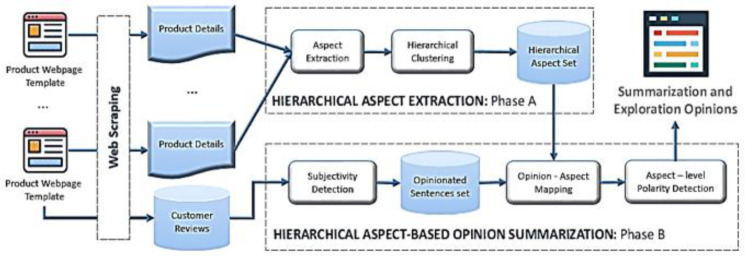
Overview of the SEOpinion system.

The general structure of our SEOpinion system is given in Figure 2. The system takes a set of product web page templates (each template has product details and customer reviews) of the same product type as input and generates a set of summaries for these products as output. The SEOpinion system is split into web scraping and two other main phases: hierarchical aspect extraction (HAE) and hierarchical aspect-based opinion summarization (HAOS). In the web-scraping phase, the product details and reviews are crawled from EC websites. After that, in the HAE phase, the popular product aspects are extracted from the product details of the same type and then stored in a hierarchical format, as shown in Figure 1b. In the HAOS phase, opinions are first extracted from reviews for each product separately. After that, these opinions are mapped to the hierarchical-aspect set. Furthermore, the polarity of each one is classified (i.e., positive or negative) according to the aspect associated with it, as shown in Figure 1d.

The proposed methodology is described using the pseudocode in Algorithm 1. The input is a set of product web page templates of the same type (P) = {p_1_, p_2_ … p_n_}, while the output is a summarization of aspects and an exploration of the gathered opinions. Step 1 initializes our system. Step 2 scraps all product details (D) and reviews (R) from P. The scraping process is encapsulated in the scraping function (Step 2). The function “HAExtraction” (Step 3) in the Algorithm creates a hierarchy of common aspects (H) from all product details in P. After that, the Algorithm iterates through all products in P (Loop 4–8). In each iteration (i.e., on each product separately), the function “HAOSummary” (Step 6) produces an aspect summary (S_i_) based on the opinions that match it. Finally, summarization aspects and exploration opinionated sentences are obtained.
**Algorithm 1.** SEOpinion System.**Input:** * P:* A set of products web page templates of the same product type = {*p*_1_*, p*_2_
*… p_n_*} **Output:**
* SEO:* Summarization and exploration opinions **Method:***1:* SEO ← φ*2:* R, D ← Scraping (P)*3:* H ← HAExtraction (D)                 ⇒ Phase A*4:* **for each** product *p_i_* ∈ *P* **do***5:*   *let R_i_ ∈ R, be a set of reviews in p_i_**6:*   *S_i_ ← HAOSummary (R_i_, H)              ⇒ Phase B**7:*   *SEO ← SEO ∪ S_i_**8:* **end for***9:* **return***SEO*

### 3.2. Web Scrapping

Web scraping is a technique used to fetch data from websites using web scrapers/crawlers. Web scrapers are scripts that use the HTTP (Hypertext Transfer Protocol) to connect to the World Wide Web (WWW) and allow users to retrieve information. Therefore, our web scraper was built to collect product information from EC websites (there are two types of information in these sites, namely product details and customer reviews). In creating the scraper, we faced three main challenges. First, generalizing websites is challenging because the templates vary from site to site. Second, web page structures are constantly updated by web developers. Hence, it is difficult to rely on a single scraper for a long time. Third, the structure of the same website may differ from one category to another. For example, on the Amazon website, the structure of the electronic category is different from the structure of the book category in the template format. To address the above-mentioned challenges, we followed the continuous development and integration of a specific domain on EC sites using the XPath query language. XPath contains the path of any element located on the web page, which is simple, powerful, concise, and easy to get. Table 1 shows the recent structure update of the extracted parts from the laptop domain on the top five EC websites using the XPath format.

**Figure 3:**
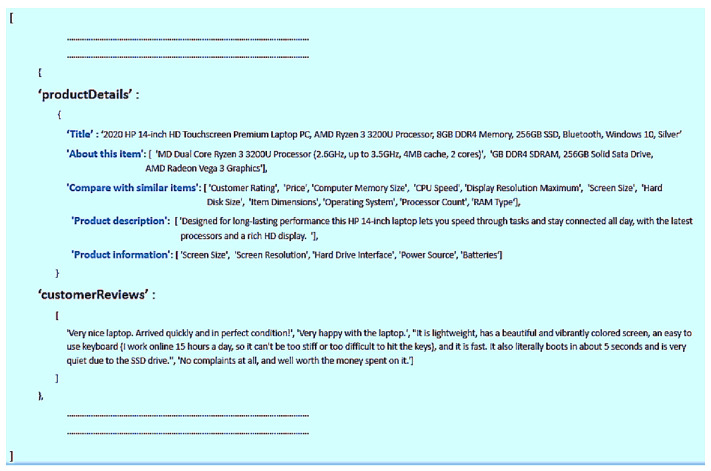
An example of a JSON (JavaScript Object Notation) object from Amazon.

The authors of this paper used the Scrapy (https://docs.scrapy.org/en/latest/) python package, as in [27,28], to create the web scraper. Scrapy was designed to scrape the web content from websites that are composed of many pages of similar semantic structures (i.e., the templates of web pages). Scrapy stands out from other scraping tools (e.g., Selenium and Apify) because it is faster, uses parallel processing, and can deal with structured data and open-sources. The steps to create our scraper are summarized as follows. First, the web scraper visits publicly available web pages that contain product details and reviews. Then, it receives HTML (Hypertext Markup Language) data back from the web server, in which the content of the web pages is embedded. After that, Scrapy extracts the useful-data parts from the HTML using the XPath format. The code snippet “response.xpath(‘XPath Format’).getall()” is used to scrape data from several EC websites using the information from Table 1 to know the XPath format structure of the top five EC websites. Finally, our scraper stores these data in a JSON (JavaScript Object Notation) file. Each JSON file contains an array of JSON objects in which each object consists of two properties (as shown in Figure 3), namely “productDetails” and “customerReviews.” On the one hand, the property “productDetails” has the element of “title” and a set of “useful-data parts,” according to each EC website. The “title” element has the name of the product itself. “Useful-data parts” have the extracted parts of the EC website, as shown in Table 1. On the other hand, the property “customerReviews” has an array of the review text.

To sum up, our scraper mainly focuses on a semi-supervised process of crawling EC websites to find product details and reviews. This step is useful to create our system because the product details are used for extracting the hierarchical aspects (the first phase). Additionally, the customer reviews are used to summarize the hierarchical aspects based on opinions (the second phase). The details of these two phases are discussed in the next two subsections.

### 3.3. Hierarchical Aspect Extraction

This section focuses on the HAE phase of our system. In this phase, popular aspects are first extracted from the product details that are gathered from EC websites. After that, these aspects are represented in different granularity levels. For example, the “camera” is an aspect of the “laptop” domain, whereas “resolution” and “lens” are components of “camera” and not of “laptop” directly. This phase is implemented in two sequential tasks named (i) aspect extraction and (ii) hierarchical clustering.

#### 3.3.1. Aspect Extraction

This task fetches the aspects from the product information (i.e., the template of the websites). It has the following advantages. First, templates have attributes that may not be mentioned by reviewers in their opinions, so this task can avoid problems of not considering important aspects. Second, the templates are provided by manufacturers, so the text does not suffer from problems with spelling, punctuation, and grammatical errors, contrary to the reviews’ comments. Finally, the manufacturers highlight the most useful product characteristics of their websites.

**Figure 4:**
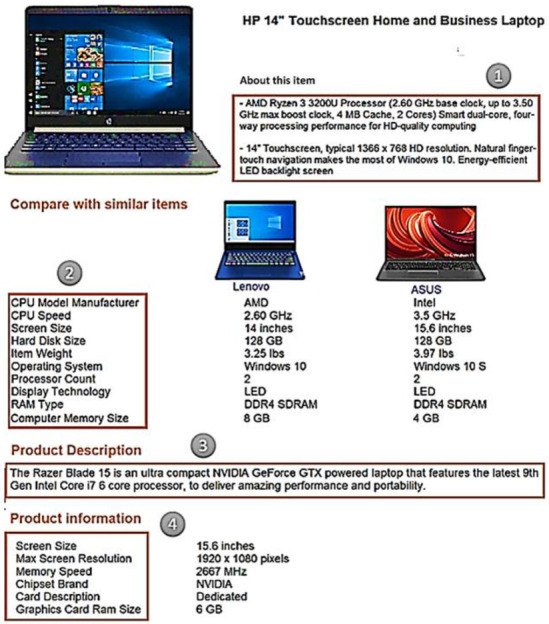
Example product details provided by manufacturers.

To extract product aspects, there are two specific types in the product template: direct and indirect aspects. The first type is represented in the first column of the <Table> tag from the EC web-source using the proposed method by [29]. The second type is found inside some paragraph texts and needs a processing to be extracted. For example, Figure 4 shows the useful data parts of the Amazon template that can be represented in four parts: About-This-Item, Product-Description, Compare-with-Similar-Items, and Product-Information. After scraping the data in the previous phase, we note that the paragraph texts appear in the first two parts because the aspects inside them are not direct and need a processing unlike direct-aspect set (Ad). The steps for extracting the indirect-aspects (Ai) are shown in Algorithm 2.
**Algorithm 2.** Hierarchical Aspect Extraction (Phase A).**Input:** *D*: A set of products details of the same product type ∈ P *θ*: Threshold score for aspect clustering **Output:** *H:* A hierarchical aspect set **Method:** *//Task 1. Aspect Extraction**1:* Direct aspect set Ad← φ, Candidate aspect set Ac← φ*2:* **for all***product details d_i_ ∈ D **do****3:*  *Ad_i_ ← Parsing (p_i_)**4:*  *Ad ← Ad ∪ Ad_i_**5:*  **for all** sentence s*_j_* ∈ *p_i_* **do***6:*   *T_i, j_ ← POS (s_j_)**7:*   *Ac_i, j_ ← ExtractNouns (T_i, j_)**8:*   *Ac ← Ac ∪ Ac_i, j_**9:*  **end for***10:* **end for***11:* A ← SemanticSimilarity (Ad, Ac) + Ad
*//Task 2. Hierarchical Clustering*
*12:* **let** each aspect *a_i_* ∈ *A* is a cluster *c_i_* ∈ *C**13:* **for all** aspect *a_i_* ∈ *A* **do***14:*  **for all** *c_j_* ∈ *C* **do**15:  **if** *ClusterSim (a_i_, c_j_) > θ ***then**16:     *c_j_ = c_j_ ∪ a_i_*
*17:*    **end if***18:*  **end for***19:* **end for***20:* update *C* with distinct clusters21:**for all***c_j_* ∈ *C* **do***22:*  **for all** aspect a*_i_* ∈ *c_j_* **do***23:*   **for all** aspect a*_k_* ∈ *c_j_* **do***24:*    **if** *ClusterSim* (*a_i_, a_k_*) be maximum **then***25:*     parent aspect is *a_i_* and reminder aspects are children, and update *H.**26:*    **end if***27:*   **end for***28:*  **end for***29:* **end for***30:* **return***H*

**Figure 5:**
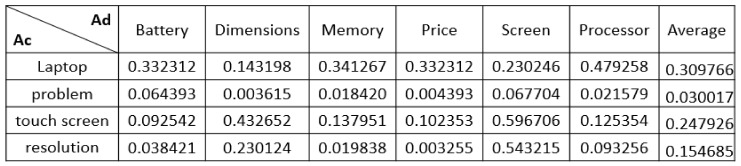
An example of semantic similarity. Ac: candidate aspects; Ad: direct aspects.

Algorithm 2 takes products details (D) of the same type (P) as input and generates as output the hierarchical aspect set (H). This algorithm is represented in two tasks: aspect extraction and hierarchical clustering. For the first task, it is devoted to extracting popular aspects (A) (Steps 1–11). Step 1 initializes the direct aspect set (Ad) and the candidate aspect set (Ac) as empty. The algorithm iterates through the product details d_i_ ∈ D (Loop 2–10), and for each one, direct aspects can be extracted using a simple parsing step, as done by [29] (Step 3), and are added to the Ad set of P (Step 4). The algorithm also iterates through the sentences s_j_ ∈ p_i_ (Loop 5–9), in which each sentence (s_j_) produces the part-of-speech tag (T_i, j_) for each word using the Stanford Log-Linear Part-Of-Speech (POS) Tagger (https://nlp.stanford.edu/software/tagger.shtml) [30] (Step 6). Step 7 selects noun words and noun phrases (T_i, j_) and identify them as candidate aspect (Ac_i, j_) to be added to the set of candidate aspects (Ac) of P (Step 8). Finally, in step 11, the popular aspects (A) are extracted by measuring the similarity between the two sets, candidate aspects (Ac) and direct aspects (Ad), using the “SemanticSimilarity” function. This function implements word embedding, which has shown great results in handling the semantic similarity, as in [31]. For example, the customer review “This laptop runs fast, but the only problem that the touch screen was not responsive and the resolution is amazing.” has four candidate aspects (Ac) represented by three nouns (“laptop,” “problem,” and “resolution”) and one noun phrase (“touch screen”). Additionally, the direct aspects are extracted for the laptop webpage as follows (“battery,” “dimensions,” “memory,” “price,” “screen,” and “processor”). For every two features, the semantic similarity is calculated using word2vec (https://code.google.com/archive/p/word2vec/) to learn word embedding. Finally, the highest averages for all candidate aspects (i.e., more than 0.1) are extracted as popular aspects. As shown in Figure 5, the popular aspects are direct aspects with the candidate aspects which average more than 0.1, such as laptop, touch screen, and resolution.

#### 3.3.2. Hierarchical Clustering

**Figure 6:**
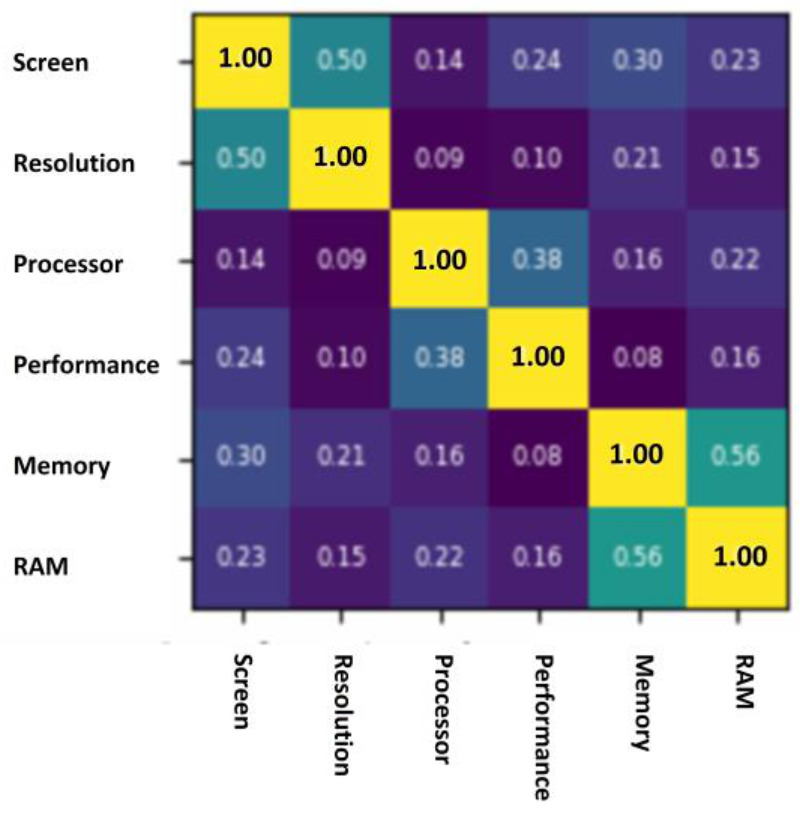
An example of lexical similarity.

This task clusters a set of product aspects into feature grouping. For example, “resolution,” “screen-size,” and “screen” are not synonymous, but they can indicate the same feature: the screen. The task applies cluster similarity technique-based an unsupervised method, which is encapsulated in the “ClusterSim” function. This function is based on the work of [32], and we mainly used lexical (or WordNet) similarity to extract a more distinct distributive context that exploits a portion of natural language knowledge to aid in clustering. As shown in Figure 6, “screen” and “resolution” have a 50% of lexical similarity; this percentage should be close because both aspects share many vocabularies. Additionally, “processor” is the closest to “performance,” and “memory” is the closest to “RAM”.

Algorithm 2 (Steps 12–30) shows the steps to obtain the hierarchical aspect set (H) given the popular product aspects (A) extracted previously (Steps 12–30). Step 12 makes each aspect a_i_ ∈ A a cluster ci ∈ C. The algorithm iterates through a set of aspects a_i_ ∈ A (Loop 13–19) and through a set of clusters c_j_ ∈ C (Loop 14–18). For each iteration, if the average similarity between the aspect (a_i_) and the cluster (c_j_) is more than the threshold score (q) (which is chosen based on its value in [32]), as in step 15, then a_i_ is merged with c_j_ (Step 16) and C is updated with distinct clusters (Step 20). For illustration, in the above example, the cluster (screen and resolution) and the aspect (memory) have 30% and 21% lexical similarities, respectively. When calculating the similarity of the aspect with the cluster, we find that the aspect achieves (30% + 21%)/2 = 25.5% (i.e., less than 35%), so it is not closest to the cluster. After that, the algorithm repeats a set of clusters c_j_ ∈ C (Loop 21–29), a set of aspects a_i_ ∈ c_j_ (Loop 22–28), and a set of aspects a_k_ ∈ c_j_ (Loop 23–27). In each iteration, if the average similarity between each pair of aspects (a_i_ and a_k_) is the maximum (Step 24), then a_i_ is a parent aspect. Otherwise, the others are children and finally update the hierarchy (H) (Step 25) to be returned in Step 30. For example, if we have a cluster (consisting of dimensions, weight, and size), then the average between them means that the “dimensions” aspect is the highest, thus making it the parent aspect.

### 3.4. Hierarchical Aspect-Based Opinion Summarization

In SEOpinion, the second phase is introduced to provide a hierarchical-aspect set (H) extracted from the previous phase with a set of opinionated sentences (O_i_), where each sentence is classified according to the polarity of its aspects associated in H. This phase is organized into three sequential subtasks, named (i) subjectivity detection, (ii) opinion mapping, and (iii) aspect-level polarity detection. These tasks are shown in Algorithm 3, which takes the hierarchical aspect set (H) and a set of reviews (R_i_) for each product (p_i_) as input and generates a hierarchical aspect-based opinion summary (S_i_), which is also repeated on all products in P.
**Algorithm 3.** Hierarchical Aspect-Based Opinion Summarization (Phase B).**Input:** *H:* A hierarchical aspect set *R_i_:* A set of reviews from a given product *p_i_* ∈ *P* **Output:** *S_i_:* A hierarchical aspect-based summary ∈ *p_i_* **Method:** *//Task 1. Subjectivity Classification**1:* opinion sentences set *O_i_* *←* *φ,* mapped aspect set *M_i_* *←* *φ,* hierarchical aspect-based summary *S_i_←* *φ**2:* **for each** review *r* ∈ *R_i_* **do***3:*  *S_T_ ← {sentence s | s ∈ r ∧ POS (s)}**4:*  **if** *S_T_ has NN and ADJ* **then***5:*   *P’ ← 0, N’ ← 0, U’ ← 0**6:*   **for all** word *w* ∈ *S_T_* **do***7:*    *P, N, U ← SemanticScore (w)**8:*    *P’ ← P’ + P**9:*    *N’ ← N’ + N**10:*    *U’ = U’+U**11:*   **end for***12:*   **if** *P’ + N’ > U’* **then***13:*     *O_i_ ← O_i_ ∪ S_T_**14:*   **end if***15:*  **end if***16:* **end if**
*//Task 2. Aspect Mapping*
*17:* $\;I_{i}^{+}$*18:* **for all** parent aspect *Ap_j_* ∈ *H* **do***19:* **let** opinion sentence *o* ∈ *O_i_**20:*  **if** *mapped (Ap_j_, o) ← true* **then***21:*   **for all** child aspect *Ac_k_* ∈ *Ap_j_* **do***22:*    **if** *mapped (Ac_k_, o) ← true* **then***23:*       *M_i_ ← M_i_ ∪ <Ac_k_, o> **24:*    **end if***25:*   **end for***26:*  **end if***27:* **end for**
*//Task 3. Aspect-level Sentiment Classification*
*28:* **for all** m_j_ ∈ *M_i_* **do***29:*  *P_j_ ← classifyPolarity (m_j_)**30:*  *S_i_← S_i_ ∪ P_j_**31:* **end for***32:* **return***S_i_*

#### 3.4.1. Subjectivity Detection

This task is often called an opinion extraction, which is used to differentiate sentences that state opinions from those that state facts. To address this task, our approach assumes that an opinion sentence (O_i_) in a review (R_i_) must include at least one noun and one adjective. Consequently, it includes three steps: Part Of Speech (POS) tagging, sentence filtering, and word-based semantic scoring.

**Figure 7:**
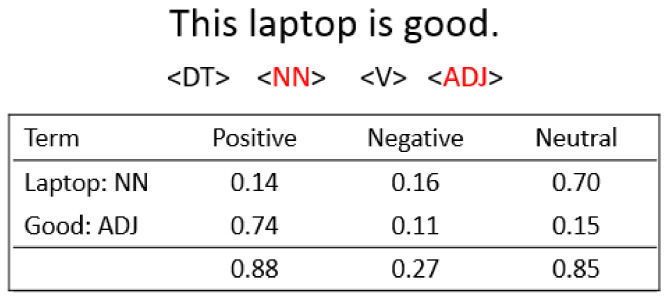
An example for subjectivity detection. DT: determiner; NN: noun; V: verb; ADJ: adjective;

As shown in Algorithm 3, this task extracts a set of opinionated sentences (O_i_) from reviews (R_i_) of a specific product p_i_ ∈ P. The algorithm iterates through review r ∈ R_i_ (Loop 2–16). In Step 3, the POS function determines part-of-speech tagging for each sentence s ∈ r using the Stanford Log-Linear POS Tagger [30]. The set of these tagged sentences is designed as S_T_. Step 4 checks if the tagged sentence (S_T_) has at least one noun (NN) and one adjective (ADJ), and then a semantic score gives a measure of the number of objective and subjective words in the sentence (Loop 6–11), which is encapsulated in the “SemanticScore” function (Step 7). This function is adopted using SENTIWORDNET [33], where each word contains positive (P), neutral (U), and negative (N) scores for each one to determine subjective words in the sentence. The positive, negative, and neutral scores are summed over all noun and adjective words in the sentence (Steps 8–10) and used to normalize the individual scores for each one. If the sum of a positive and negative scores larger than the neutral score (Step 12), then the sentence is defined as an opinionated/subjectivity sentence. Figure 7 shows an example of computing the normalized score over a noun “laptop” and an adjective “good.” The sentence is subjectivity, in which “good” achieves (0.88 + 0.27) > 0.85. These steps are performed on all sentences of each review to construct a full set of opinion sentences (O_i_) for the specific product (p_i_) (Step 13).

#### 3.4.2. Aspect/Opinion Mapping

This task maps opinionated sentences according to the specific aspects that have been given by manufacturers. Hence, there are two cases for this task in our system: (i) the opinion may indicate the aspect category as a whole (general) or (ii) one of the aspect terms (child aspects) of the aspect category. Proposals in [34] demonstrated the mapping of opinions in one level aspect. In contrast, our approach maps the opinionated sentences (*O_i_*) according to their aspects in two-levels of *H*.

As shown in Algorithm 3, this task receives a hierarchical aspect set (H) and opinionated sentences (O_i_) = {o_1_, o_2_ … o_n_}, which were extracted from the previous section, and returns a mapped aspect set (M_i_) (Steps 17–27). The algorithm iterates through the set of parent aspects (Ap) inside the hierarchical aspect set (H) (Loop 18–27). In Step 20, if a parent aspect (Ap_j_) is mapped to an opinionated sentence (o) as applied in [34], the algorithm also repeats the process with the child aspects (Ac) associated with its parent aspect (Loop 21–25). In each iteration, if a child aspect (Ac_k_) is mapped to an opinion sentence (o) (Step 22), it stores a mapping aspect set (M_i_) in pairs of the child aspect (Ac_k_) and its opinion (o) (Step 23).

#### 3.4.3. Aspect-Level Polarity Detection

The main subtasks of sentiment classification are emotion identification [35], sentiment intensity prediction [36], and polarity detection [37]. Emotion identification detects the emotions behind sentiments such as anger or sadness. Predicting sentiment intensity seeks to identify the polarity degree (e.g., ‘good,’ ‘wonderful,’ and ‘awesome’). Furthermore, polarity detection classifies text as negative or positive. Hence, our system applies the polarity detection task to the aspect, which classifies polarity for each opinionated sentence based on the aspect that matches it. Polarity detection has been solved using different machine learning techniques [8,38]. Recently, DL techniques have achieved success in polarity detection [38], especially with the use of BERT embedding. Our work uses DL-based BERT representation, and the results showed that combination of BERT with DL methods worked well for this task [39].

**Figure 8:**
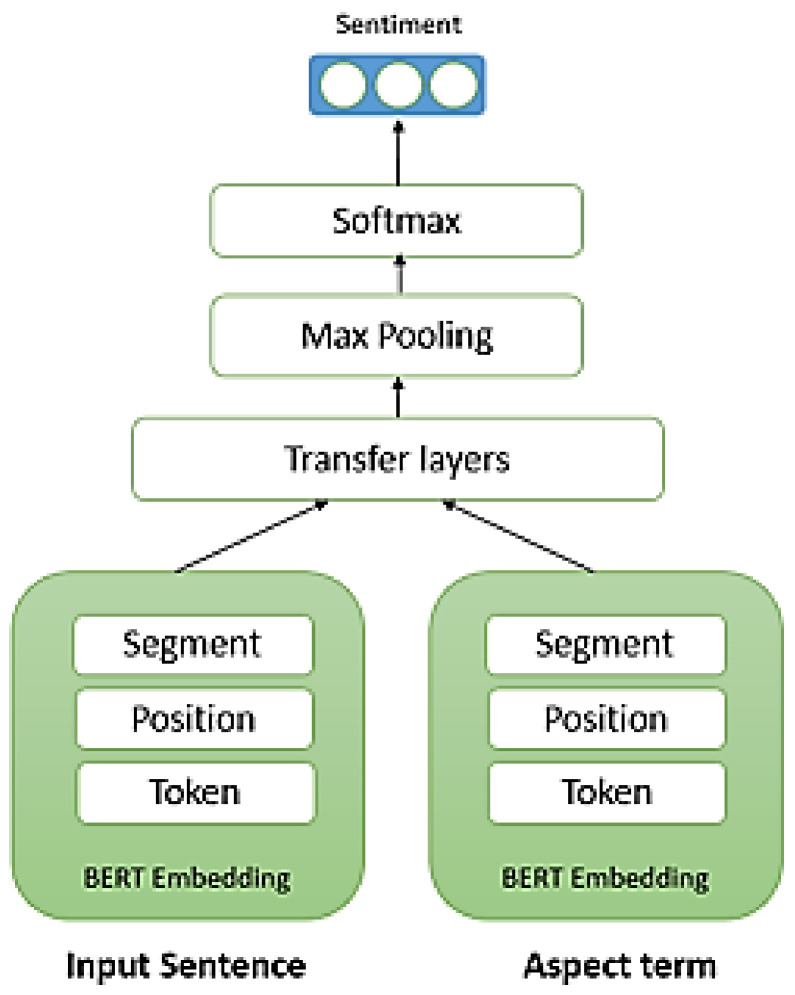
The proposed architecture.

BERT (Bidirectional Encoder Representations from Transformers) is one of the major shifts in new advances in learning contextual representation as more information is provided [5], and it has been widely implemented in sentiment analysis tasks [39]. Our model is shown in Figure 8. As can be seen, the BERT embedding layer takes an aspect and a sentence as input and computes the token-layer feature level and outputs a positive, negative, or neutral class. Additionally, we integrate three different embeddings corresponding to the input token: token, position, and segment. Token embedding is a vector representation of each token in the vocabulary. Position embeddings are applied to protect information about the placement of words in a sentence. Segment embeddings are adopted to recognize between sentences. After that, the transformer layers are inserted to optimize the token level features layer by layer. After the input passes through the network, in the last layer, emotions are extracted by applying fully connected layers to their encoder.

Algorithm 3 receives a map aspect set (M_i_) from the previous task and returns a hierarchical aspect-based opinion summary (S_i_) (Steps 28–32). The algorithm iterates through the set of the mapped aspect set (m_i_) (Loop 28–31). For each iteration, in step 29, the task classifies the polarity of opinion (positive or negative) according to its child aspect, as in [39], using the “classifyPolarity” function. In step 30, the polarity (P_i_) extracted from this function is added to the summary (S_i_). Finally, it generates the hierarchical aspect-based summary S_i_ ∈ p_i_, which contains a set of aspect terms associated with their classified sentences (Step 32), as shown in Figure 1d.

#### 3.5. User Interface

**Figure 9:**
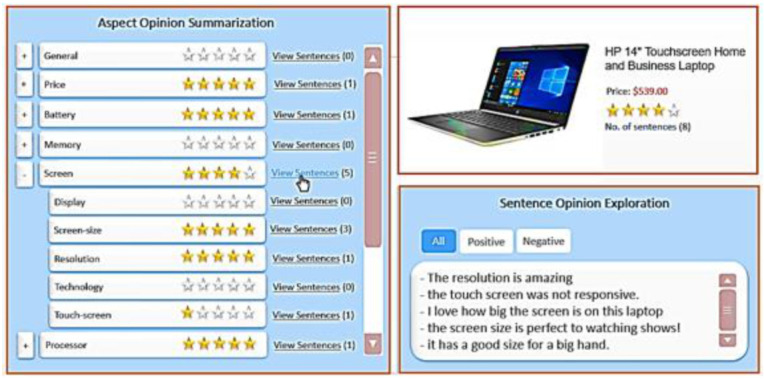
Screenshot of our SEOpinion system.

Our system introduces a comparison among a set of products of the same type by providing a summarization and an exploration interface, as shown in Figure 9. This interface enables a user to browse through several product aspects and go through related opinions. Figure 9 displays a screenshot of the SEOpinion interface showing information related to “laptop.” The interface consists of three panels, namely (i) the product presentation panel, (ii) the aspect-opinion-summarization panel, and (iii) the sentence-opinion-exploration panel.

1. The product presentation panel shows information about the product, such as its name, price, images, rate summary of its opinion sentences, and the number of these sentences in the top-level aspects (e.g., general, price, battery, memory, screen, and processor).
2. The summarization panel displays the hierarchy aspects of the product and the aspect-based summary. Initially, sub-aspects are kept hidden until the user clicks on the related parent aspect. For example, “display,” “screen-size,” “resolution,” “technology,” and “touch-screen” are components or sub-aspects of the “screen.” For each top-level aspect, the total of sentences on each aspect is shown because it gives other users the confidence of the aspect rate (i.e., when the number of sentences increases, the user’s confidence in the rating aspect increases). Furthermore, the rated summary for each aspect is the average for the scores of its sentences. Our system considers positive = 5 and negative = 1. For example, as shown in Figure 9, the aspect “screen” contains five sub-aspects for five sentences, which include four positive and one negative. Thus, the average of all sentences for summarizing the aspect “screen” is calculated as (5 + 5 + 5 + 5 + 1)/5 = 4.2.
3. The exploration panel shows the opinionated sentences that are categorized as positive or negative. Initially, this panel does not display these sentences of the product if no product aspect is selected. These sentences related to the aspect are shown in this panel only when the user clicks on the “view sentences” button of the aspect in the summarization panel.

This way, the user is not distracted with irrelevant aspects. The valued-added feature of this interface is that it allows an interaction between users and content, which makes it easy for an end-user to identify product aspects of interest and focus on sentences that contain relevant aspect information.

## 4. Experiments

This section describes several experiments that were conducted to evaluate the performance of our SEOpinion system. First, the datasets and the preprocessing steps are presented. Second, the baseline methods are introduced. Third, the evaluation metrics are described for the proposed approach, and, finally, the experiment setups are depicted.

### 4.1. Data Collection and Preprocessing

A collection of products/items from the same product type (e.g., book or camera) on EC websites was needed to show the effectiveness of the proposed approach. Each website contains product details identified with aspect categories and aspect terms (as shown in Figure 1b), and review sentences are labeled with aspects and polarities/sentiments (as shown in Figure 1d). As there was no such benchmark corpus, we created a laptop collection from five EC websites (LC5) dataset crawled from Amazon, Flipkart, eBay, Walmart*,* and BestBuy. This dataset will be available publicly for researchers. On each item, the aspect terms are manually fetched from product details and the sentences of reviews are annotated to the extracted aspects. Each sentence belonging to one aspect is also labeled as expressing a positive or negative sentiment (ignoring the scores of neutral, since it is not useful for the aspect summarization). Details of the dataset are shown in Table 2, which shows the distribution of aspect terms, aspect categories, and sentences with polarity for each one product item.

Customer reviews are usually unstructured, full of noise, have spelling errors, are arbitrary, short, and have incomplete grammatical structures due to the frequent presence of irregular grammar, malformed words, acronyms, non-dictionary terms, etc. These factors are the most common problems in customer reviews and affect the performance of sentiment analysis tasks. Therefore, the preprocessing of our LC5 dataset was performed by removing all numbers, stop words, all non-ASCII and English characters, and all URL links. This was followed by replacing negative references, emoticons, and slang with their full word forms and expanding acronyms. Finally, the Natural Language Toolkit (NLTK) [40] was adopted for tokenization. After being preprocessed, the LC5 dataset was ready for testing our approach.

### 4.2. Baseline

The experiments were conducted with numerous baseline methods that can be split into the two following categories:

1. Traditional machine learning: The SVM classifier is a state-of-the-art traditional machine learning method that exploits input features such as uni/bigram features and POS tags, as in [41], where the authors performed rapid dropout training by sampling or combining a Gaussian approximation. These measures were justified by central boundary theory and empirical evidence [41].
2. Deep learning: CNN and RNN utilize word embedding as an input feature, in which the embedding is trained using random initialization, Global Vectors (GloVe) [42], and BERT embedding [5]. We used GloVe instead of Word2vec because it achieved better results [38]. The pre-train BERT word embedding [5] was used on the Amazon corpus.

### 4.3. Evaluation Measures

The baseline methods were evaluated on the two main phases of our system: (i) hierarchical aspect extraction and (ii) hierarchical aspect-based opinion summarization. In both, the performance was measured using the metrics of recall (R), precision (P), and F1-measure (F). These metrics were calculated through the confusion matrix, as shown in Table 3. The table shows a confusion matrix for two classes (positive and negative), where TP (true positive) means a positive observation that is predicted as positive and TN (true negative) means a positive one that is predicted as negative (i.e., both are sampled correctly). In contradiction, FP (false positive) means a negative one that is predicted as negative, and finally FP (false positive) means the observation is negative and is predicted as positive (i.e., both are incorrect). These metrics are shown in Equations (1)–(3), which are commonly used for sentiment analysis performance. Recall measures the percentage of labels found by the system. Precision measures the percentage of labels correctly assigned by the system. The F1-measure is based on precision and recall for presenting the right results. From another perspective, the baselines are evaluated for each task in the two main phases through an accuracy metric. Accuracy represents the correct results, as shown in Equation (4), which is commonly used to measure the performance of sentiment classification approaches.

(1) $$\mathrm{Recall}\;=\;\frac{TP}{TP+FN}$$

(2) $$\mathrm{Precision}\;=\;\frac{TP}{TP+FP}$$

(3) $$F1-\mathrm{measure}\;=\;\frac{2\;*\;Precision\;*\;Recall}{Precision\;+\;Recall}$$

(4) $$\mathrm{Accuracy}\;=\;\frac{TP+TN}{TP+TN+FP+FN}$$

### 4.4. Experiment Setups

Our experiments tuned the settings of the CNN and RNN models, where these models were implemented in the PyTorch [43] framework (https://pytorch.org/) and on a single NVIDIA Tesla P100 GPU. Additionally, the two models were applied to two types of embedding layers: GloVe and BERT. For the learning process, GloVe embedding was used, as in [44]. In contrast, the BERT embedding was fine-tuned to keep the dropout probability at 0.1 [5]. Additionally, an Adam optimizer was used to update the model parameters [44]. The best hyper-parameter batch size and learning rate were obtained as {16,32} and {2 × 10^−5^, 3 × 10^−5^}, respectively, by grid search. Finally, the details of used hyper-parameters and the configurations are shown in Table 4. For each experiment, ten-fold cross-validation was applied 100 times for each website of our dataset. Additionally, the average accuracy was obtained by observing 100 replications of cross-validation. An imbalance in our LC5 dataset was the main problem, as positive reviews were not equal to negative reviews. To address this problem, we performed a sampling of various sub-datasets and took the average of the outcomes for each one.

## 5. Experimental Results and Analysis

The objective or our analysis was to comprehensively evaluate the performance of the SEOpinion system in the above two sequential phases: HAE and HAOS. The results were analyzed and achieved by SEOpinion when applied to our LC5 dataset. The results of HAE and HAOS are displayed in Table 5 and Table 6, respectively. These results were achieved on our LC5 dataset and are shown in three parts: in part I, SVM was based on the traditional feature-engineering method [45]. Parts II and III contained deep learning methods (CNN and RNN) based on word embedding, including pre-trained word vectors (random initialization, GloVe [42], and BERT [5]).

### 5.1. Results for Hierarchical Aspect Extraction

This stage uses two sequential tasks for creating a hierarchical aspect set, such as aspect extraction and hierarchical aspect clustering. Table 5 shows our results for the HAE phase on the LC5 dataset. As can be seen from the table, the SVM method had the worst performance for the F-measure of the five product websites. In contrast, the RNN method with BERT embedding was the best, as the highest F1-measure was 80.9% for Amazon and the minimal F1-measure was 73.3% for eBay. Moreover, the F1-measure of the RNN and BERT outperformed the SVM, CNN, CNN and GloVe, RNN, and RNN and GloVe groups for Amazon, eBay, Walmart, and BestBuy. The RNN and BERT group had similar results as RNN and GloVe on all websites of our dataset.

### 5.2. Results for Hierarchical Aspect-Based Opinion Summarization

This stage uses the three sequential tasks for summarizing the extracted hierarchical aspect set by opinions, such as subjectivity classification, opinion mapping, and aspect-level polarity detection. Table 6 displays our results for the HAOS phase on our LC5 dataset. As can be seen from the table, the SVM method had the worst performance for the F1-measure on all EC websites because it used the feature extraction of sentiment analysis tasks. On the other hand, the RNN method with BERT embedding was the best, as the highest F1-measure was 86.0% for Amazon and the minimal F1-measure was 78.7% for Flipkart. Moreover, the F1-measure of the RNN and BERT group outperformed the SVM, CNN, CNN and GloVe, RNN, and RNN and GloVe groups on four out of five websites. The RNN and BERT group had similar results as the RNN and GloVe group for Flipkart.

### 5.3. Analysis of Results

**Figure 10:**
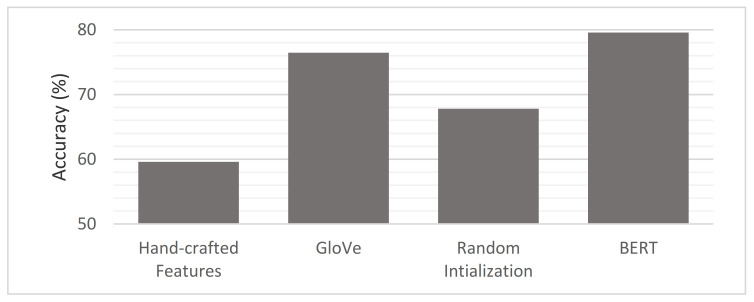
Accuracies from the four representation types.

**Figure 11:**
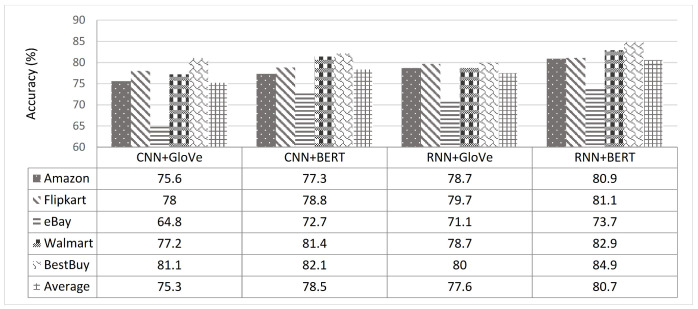
Comparisons of the two word embedding-based deep learning models for our LC5 dataset.

**Figure 12:**
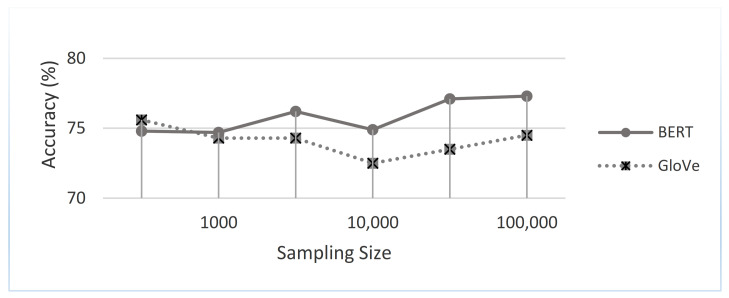
Accuracies from the sampling of different sizes for our dataset.

**Figure 13:**
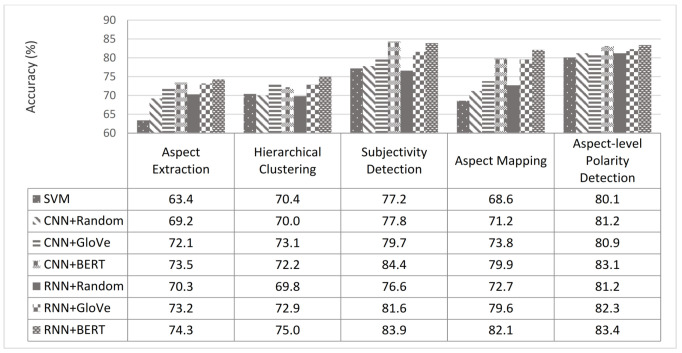
Comparisons of the baseline models for the five research tasks.

**Figure 14:**
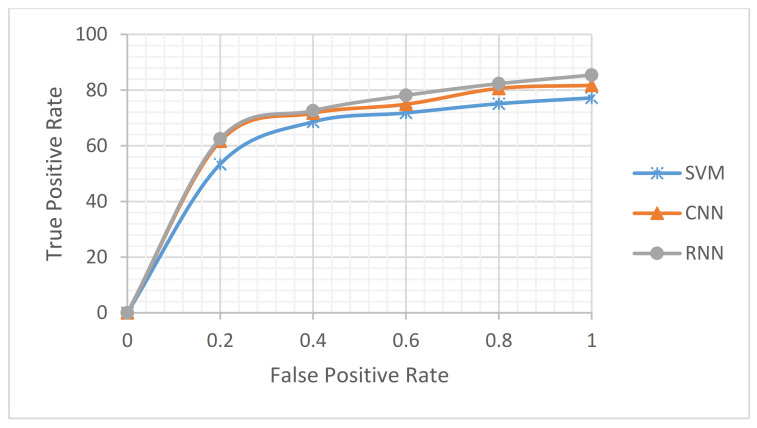
Area under the ROC curve on LC5 dataset with (area = 0.71).

From another perspective, through an accuracy metric, we studied which text representations achieved the best results. The impact of the four different representations—namely the hand-crafted features [45], GloVe embeddings [42], random initialization embeddings, and BERT embeddings [5]—on our proposed approach was investigated. Figure 10 shows the performance of the four different representations on our LC5 dataset. The GloVe embedding and BERT embedding-based deep learning models (i.e., RNN and CNN) were studied and achieved the best results. Therefore, a comparison of the two words embedding-based DL models (i.e., CNN and RNN) on both phases revealed the average performance for each product website, as shown in Figure 11. As can be seen, the RNN-based BERT method had the highest accuracy among all websites, and the CNN based-GloVe method had the lowest accuracy among all websites. More generally, on both RNN and CNN, the BERT method increased the accuracy of the GloVe method by an approximate average of at least 3.1%. Additionally, the accuracy difference between GloVe embedding and BERT embedding increased when the volume of data grew, such as with the BestBuy, Amazon, and Walmart websites. To study the size of the data in depth, Figure 12 shows the overall picture of BERT embedding and GloVe embedding on the average of both phases. Note that the BERT embedding achieved the best results for large data sizes. In contrast, GloVe embedding had the best accuracy rates obtained for small data volumes. More generally, when studying the baseline model performance, we looked at the five tasks of our system (aspect extraction, hierarchical clustering, subjectivity detection, opinion mapping, and aspect level-polarity detection) separately. As shown in Figure 13, the BERT embedding-based two DL models provided a significant increase in accuracy metric over GloVe embedding while also being noticeably superior to other baselines on different tasks. Additionally, the aspect extraction and hierarchical clustering tasks were the least accurate in comparison to others due to the small amount of retrieved information from product template websites. Thus, our intention in the future is to try the proposed approach on another domain (movies or restaurants). Comparing the results in Table 5 and Table 6 reveals that BERT was an effective model for predicting sentiment in the LC5 dataset. In Figure 14, we compare the area under the ROC (Receiver Operating Characteristic) curve for the results of applying SVM, CNN, and RNN.

To sum up, from the presented results, we can observe that DL techniques were more flexible than the SVM approach with hand-crafted features. Furthermore, the BERT embedding showed significance in the performance of the DL models, especially the RNN model. Additionally, it was found that BERT could benefit from a larger training set than GloVe.

## 6. Limitations and Future Directions

The conducted experiments achieved the best results with the RNN-based BERT approach. However, our results were not the best among the existing summarization systems [13] because they ignored the multi-granularity of aspects and used pre-defined aspects. Moreover, our approach has several limitations that could be summarized as follows:

1. The system only works well with web pages that have many product details (aspects) embedded in the page’ templates. Therefore, the worst result was for the eBay website, as shown in Figure 11, because that template had fewer details in its webpage templates than others.
2. Unlike reviews, implicit aspects are difficult to be extracted from a template. In [47], a rule-based approach to obtain both explicit and implicit aspects from customer reviews was proposed.
3. The opinion mapping task in our system worked on matching an opinion sentence with only one aspect. Some opinion sentences may express more than one aspect. For example, the opinion sentence “my phone is good for its price and performance” is associated with two aspects: “price” and “performance.”

For future work, we plan to investigate strategies for improving the performance of our system as follows: The first strategy is using multichannel embedding [48] on various DL models, such as RNN and CNN, where the pre-trained word embeddings are directly incorporated into the word embedding matrix. The advantages of multichannel embedding are that it can provide rich semantic/sentiment representations and avoid word embedding interference. Thus, our expectation is that multichannel embedding will give better results than single embedding. The second strategy is potentially improving the aspect extraction task by incorporating aspects obtained from a template with the other aspects (explicitly or implicitly) mentioned in customer reviews.

## 7. Conclusions

This paper investigated the effectiveness of the BERT embedding component with the CNN and RNN models for creating our SEOpinion system. SEOpinion compares a set of products in EC websites from the same type on two phases: (i) HAE and (ii) HAOS. Hence, the experimental results demonstrated the superiority of the BERT embedding-based RNN and CNN model in both phases, as it was better than GloVe embedding by up to 3.1% on the LC5 dataset. Moreover, the results showed that the RNN-based BERT model achieved an impressive performance on a variety of LC5 benchmark datasets for the opinion summarization task, while RNN achieved better results than CNN and SVM, which reached averages of 77.4% and 82.6% in terms of F1 measure for the HAE and HAOS phases, respectively. Our system only focused on comparing a group of products from the same type in terms of summarizing the aspect’s opinions. In the future, we plan to apply it to other domains (as movies or restaurants). The proposed system is expected to work well with these domains.

## Figures and Tables

**Table 1:** Samples of different structures in the top five e-commerce (EC) websites.

EC Website	Useful Data Parts	XPath Format
Amazon	Title	*//span[@id=‘productTitle’]/text()*
	About this item	*//div[@id=‘feature-bullets’]/ul/li/span/text()*
	Compare with similar items	*//table[@id=‘HLCXComparisonTable’]//tr/th/span/text()*
	Product description	*//div[@id=‘productDescription’]/text()*
	Product information	*//table[@id=‘productDetails_techSpec_section_1′]//tr/th/text()*
	Customer Reviews	*//div[@data-hook=‘review-collapsed’]/span/text()*
Flipkart	Title	*//span[@class=‘_35KyD6′]/text()*
	Highlights	*//div[@class=‘_3WHvuP’]/ul/li/text()*
	Description	*//div[@class=‘_3la3Fn _1zZOAc’]/p/text()*
	Specifications	*//table[@class=‘_3ENrHu’]/tbody/tr/td*[1]*/text()*
	Customer Reviews	*//div[@class=‘qwjRop’]/div/div/text()*
eBay	Title	*//h1[@id=‘itemTitle’]/text()*
	Item specifics	*//td[@class=‘attrLabels’]/text()*
	About this product	*//div[@class=‘prodDetailSec’]/table/tbody/tr/td*[1]*/text()*
	Review Text	*//div[@class=‘ebay-review-section-r’]/p/text()*
	Customer Reviews	*//div[@class=‘ebay-review-section-r’]/p/text()*
Walmart	Title	*//h1[@itemprop=‘name’]/text()*
	About This Item	*//div[@class=‘about-desc about-product-description xs-margin-top’]/ul/li/text()*
	Specifications	*//table[@class=‘product-specification-table table-striped’]/tbody/tr/td*[1]*/text()*
	Customer Reviews	*//div[@class=‘review-text’]/p/text()*
BestBuy	Title	*//h1[@itemprop=‘name’]/text()*
	Other Specifications	*//table[@class=‘product-spec’]/tr/td*[1]*/text()*
	Description	*//div[@itemprop=‘description’]/text()*
	Customer Reviews	*//div[@class=‘user-review’]/p/text()*

**Table 2:** Statistics of our LC5 (laptop collection from five EC websites) dataset.

Dataset (Domain)	No. Laptop Reviewed Items (Web Pages)	For Each One Item (Average)				Polarity	
		No. Aspect Terms	No. Aspect Categories	No. Sentences	No. Sentences/Aspect Term	Positive %	Negative %
Amazon	707	42	11	3289	77.3	62	38
Flipkart	284	55	8	546	6.9	69	31
eBay	856	74	3	11	0.12	73	27
Walmart	790	18	6	2180	97.1	62	38
BestBuy	525	72	17	3574	43.4	67	33

**Table 3:** Confusion matrix for two classes. TP: true positive; FP: false positive; FN: false negative; TN: true negative.

		Prediction Label	
		*Positive*	*Negative*
**Actual Label**	*Positive*	TP	FN
	*Negative*	FP	TN

**Table 4:** The details of used hyper-parameters and the configurations on deep learning-based BERT methods.

*Word Embedding*	*BERT* [5]
*Dropout Rate*	*0.1*
*Batch Size*	*Search from = {16,32}*
*Learning Rate*	*Search from = {2 × 10^−5^, 3 × 10^−5^}*
*Max Epoch*	*6*
*Max Sequence Length*	*128*
*Optimizer*	*Adam* [44]
*Embedding Layer Dimension*	768
*Deep Learning Framework*	*Pytorch* [43]

**Table 5:** Comparison results for hierarchical aspect extraction phase in our system on our LC5 dataset. SVM: support vector machine; CNN: convolutional neural network; RNN: recurrent neural network.

Model	Text Representation		Amazon			Flipkart			eBay			Walmart			BestBuy			Avgerage
			P	R	F	P	R	F	P	R	F	P	R	F	P	R	F	
SVM	Hand-Crafted Features [41]		62.4	62.5	62.4	64.0	65.0	64.5	62.4	62.5	62.4	62.3	62.1	62.2	59.4	59.5	59.4	62.2
CNN	Embedding	Random	72.4	66.0	69.1	70.3	71.0	70.6	63.2	62.6	62.9	60.5	58.9	59.7	57.1	53.0	55.0	63.5
		GloVe [46]	73.1	66.8	69.8	71.9	79.8	75.6	64.4	63.9	64.1	70.9	64	67.3	68.5	62.5	65.4	68.5
		BERT (our)	79.6	73.3	76.3	72.1	79.8	75.8	72.5	68.8	70.6	72.9	79.9	76.2	72.8	75.8	74.3	74.7
RNN		Random	72.7	72.8	72.7	74.8	75.0	74.9	73.3	73.2	73.2	71.6	73.9	72.7	76.1	72.1	74.0	73.5
		GloVe [46]	82.4	76.0	79.1	80.3	81.0	80.6	73.2	72.6	72.9	70.5	68.9	69.7	67.1	63.0	65.0	73.5
		BERT (our)	83.3	78.7	80.9	77.7	84.1	80.8	73.4	73.3	73.3	77.5	75.7	76.6	74.4	75.9	75.1	77.4

**Table 6:** Comparison results for hierarchical aspect-based opinion summarization phase in our system on our LC5 dataset. SVM: support vector machine; CNN: convolutional neural network; RNN: recurrent neural network.

Model	Text Representation		Amazon			Flipkart			eBay			Walmart			BestBuy			Avgerage
			P	R	F	P	R	F	P	R	F	P	R	F	P	R	F	
SVM	Hand-Crafted Features [41]		73.9	74.1	74.0	72.3	75.3	73.8	65.6	61.0	63.2	71.5	73.5	72.5	71.4	73.4	72.4	71.2
CNN	Embedding	Random	79.2	89.7	84.1	79.3	75.7	77.5	64.9	55.5	59.8	76.5	76.0	76.2	74.6	79.2	76.8	75.0
		GloVe [46]	82.6	78.3	80.4	80.3	78.7	79.5	73.2	72.6	72.9	77.1	63.0	69.3	79.5	84.1	81.7	76.9
		BERT (our)	83.1	88.8	85.9	80.9	74.0	77.3	78.3	77.0	77.6	78.5	72.5	75.4	81.2	83.4	82.3	79.7
RNN		Random	83.1	76.8	79.8	81.0	75.3	78.0	74.4	73.9	74.1	80.9	74.0	77.3	78.5	72.5	75.4	77.0
		GloVe [46]	84.5	81.7	83.1	81.6	78.3	79.9	82.5	78.8	80.6	78.7	75.5	77.1	80.5	75.7	78.0	79.8
		BERT (our)	86.1	85.9	86.0	76.3	79.8	78.0	84.5	85.3	84.9	80.0	77.5	78.7	82.0	88.5	85.1	82.6

## Data Availability

Not applicable.

## Abbreviations

Abbreviations The following abbreviations are used in this manuscript:

EC	E-commerce
AOS	Aspect-based Opinion Summarization
SEOpinion	Summarization and Exploration of Opinions
HAE	Hierarchical Aspect Extraction
HAOS	Hierarchical Aspect-based Opinion Summarization
BERT	Bidirectional Encoder Representations from Transformers
GloVe	Global Vectors
DL	Deep Learning
CNN	Convolutional Neural Network
RNN	Recurrent Neural Network
SVM	Support Vector Machine
LC5	Laptop Collection from five EC websites
XML	Extensible Markup Language
URL	Uniform Resource Locator
NLTK	Natural Language Toolkit
ASCII	American Standard Code for Information Interchange
ROC	Receiver Operating Characteristic
